# Supplementary material for: Chaotic, informational and synchronous behaviour of multiplex networks
Source: Sci Rep. 2016 Mar 4;6:22617. doi: 10.1038/srep22617 (PMC4778120; doi:10.1038/srep22617)
Supplement: Supplementary Information [file srep22617-s1.pdf]

Supplementary Material of ”Chaotic, informational and synchronous behaviour of multiplex networks”

M. S. Baptista[1,*,+], R. M. Szmoski[2,+], R. F. Pereira[3,+], and S. E. de Souza Pinto[4,+]

[1]Institute for Complex Systems and Mathematical Biology, SUPA, University of Aberdeen, Aberdeen, United Kingdom
[2]Department of Physics, Federal University of Technology - Paraná, 84016-210, Ponta Grossa, Paraná, Brazil
[3]Department of Mathematics, Federal University of Technology - Paraná, 84016-210, Ponta Grossa, Paraná, Brazil
[4]Departamento de Física, Universidade Estadual de Ponta Grossa, 84030-900, Paraná, Brazil
[*]murilo.baptista@abdn.ac.uk
[+]these authors contributed equally to this work

ABSTRACT

The understanding of the relationship between topology and behaviour in interconnected networks would allow to characterise and predict behaviour in many real complex networks since both are usually not simultaneously known. Most previous studies have focused on the relationship between topology and synchronisation. In this work, we provide analytical formulas that shows how topology drives complex behaviour: chaos, information, and weak or strong synchronisation; in multiplex networks with constant Jacobian. We also study this relationship numerically in multiplex networks of Hindmarsh-Rose neurons. Whereas behaviour in the analytically tractable network is a direct but not trivial consequence of the spectra of eigenvalues of the Laplacian matrix, where behaviour may strongly depend on the break of symmetry in the topology of interconnections, in Hindmarsh-Rose neural networks the nonlinear nature of the chemical synapses breaks the elegant mathematical connection between the spectra of eigenvalues of the Laplacian matrix and the behaviour of the network, creating networks whose behaviour strongly depends on the nature (chemical or electrical) of the inter synapses.

For the *symmetric* configuration of the discrete shit map network , we consider the following inequality:

$$\gamma < \frac{\varepsilon \omega_1 N_1}{2\ell_{12}}, \tag{1}$$

For the *asymmetric* configuration of the discrete shift map network, we consider the following inequality:

$$\gamma < \frac{\varepsilon \omega_1 N_1}{\ell_{12}}, \tag{2}$$

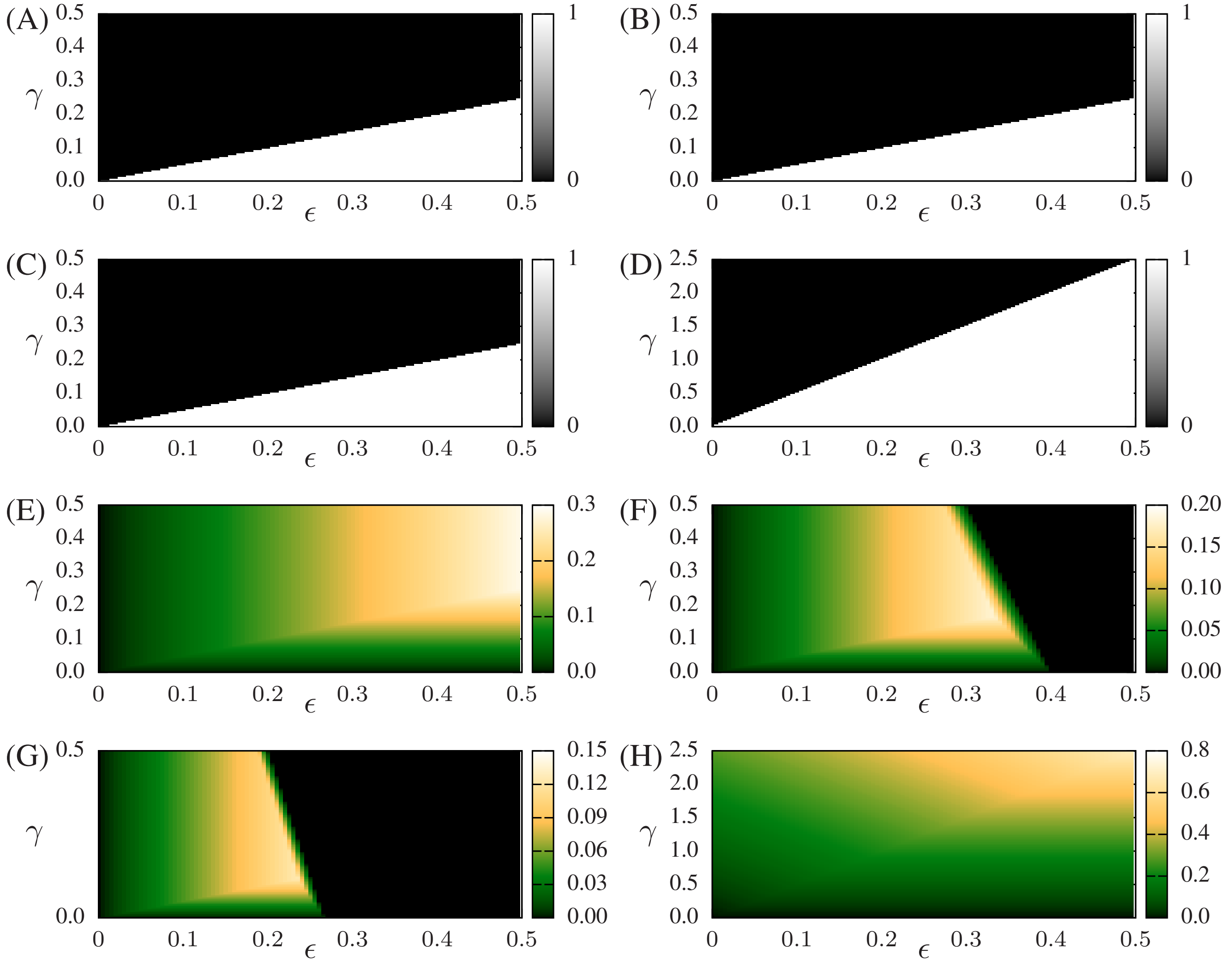

Figure 1. Two coupled stars. (A-C) White (black) region indicates values of ε and γ for which inequality (1) is satisfied (not satisfied). Colour code same as in (A-C) but based on inequality (2). (E-H) color code shows the value of I_C. N=10,ℓ_{12}=5 in (A) and (E), N=20, ℓ_{12}=10 in (B) and (F), N=30,ℓ_{12}=15 in (C) and (G), N=10, ℓ_{12}=1 in (D) and (H).

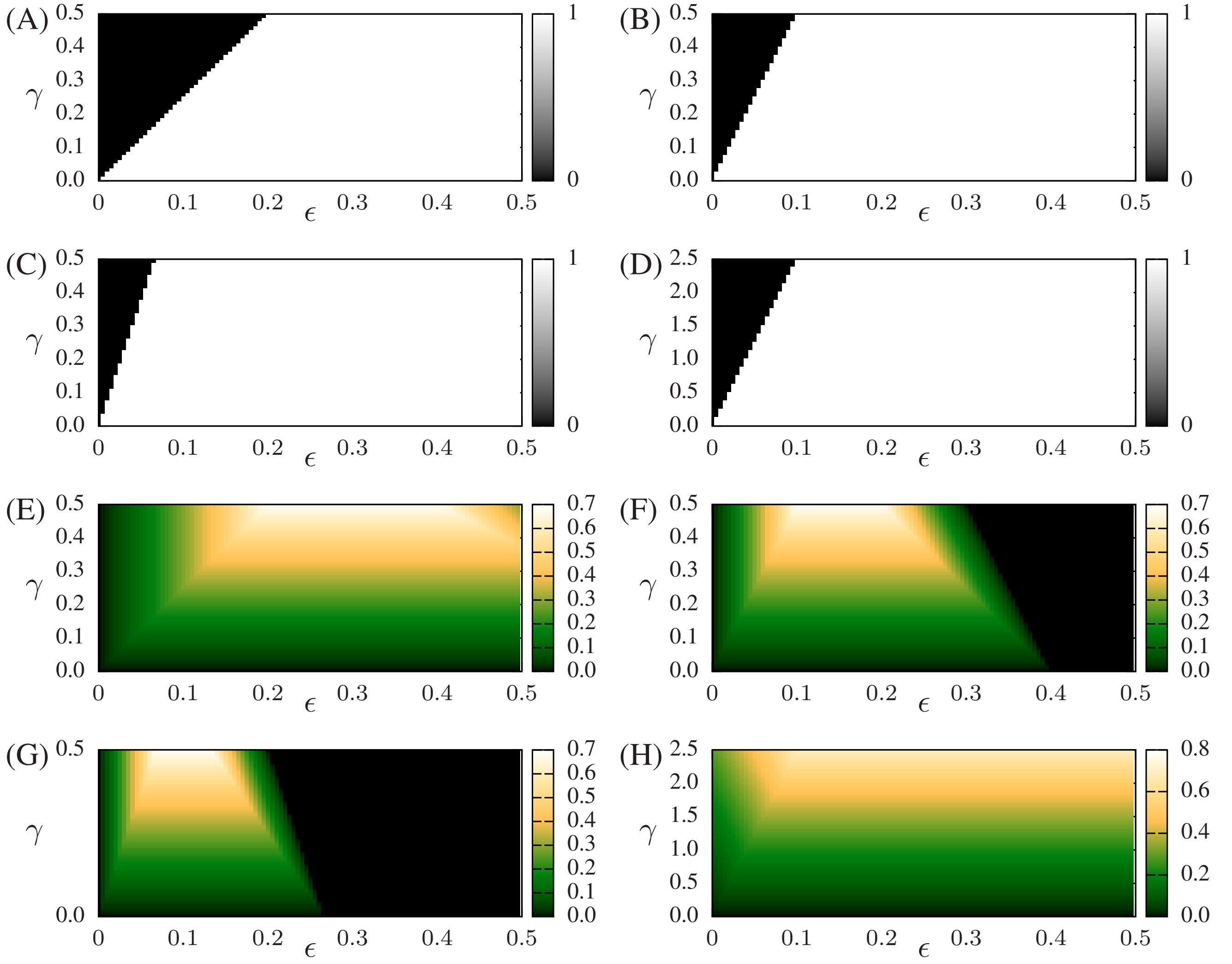

Figure 2. Two coupled all-to-all networks. (A-C) White (black) region indicates values of ε and γ for which inequality (1) is satisfied (not satisfied). Colour code same as in (A-C) but based on inequality (2). (E-H) color code shows the value of I_C. N=10,ℓ_{12}=5 in (A) and (E), N=20, ℓ_{12}=10 in (B) and (F), N=30,ℓ_{12}=15 in (C) and (G), N=10, ℓ_{12}=1 in (D) and (H).

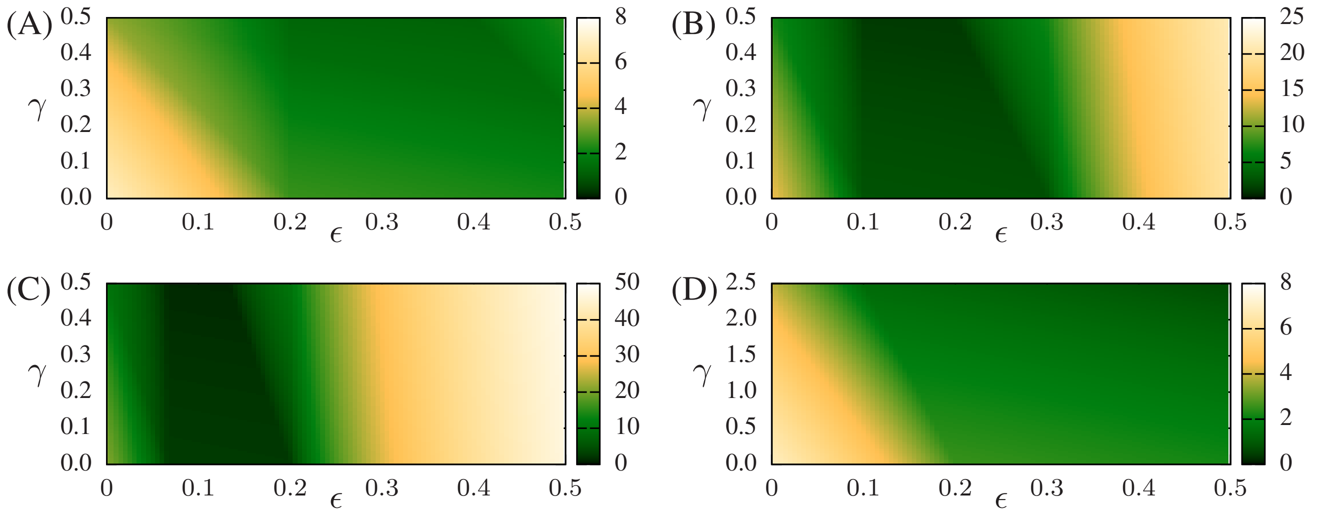

Figure 3. Sum of positive Lyapunov exponents for a network constructed by coupling two star subnetworks. N=10 and ℓ_{12}=5 in (A), N=20 and ℓ_{12}=10 in (B), N=30 and ℓ_{12}=15 in (C), and N=10 and ℓ_{12}=1 in (D).

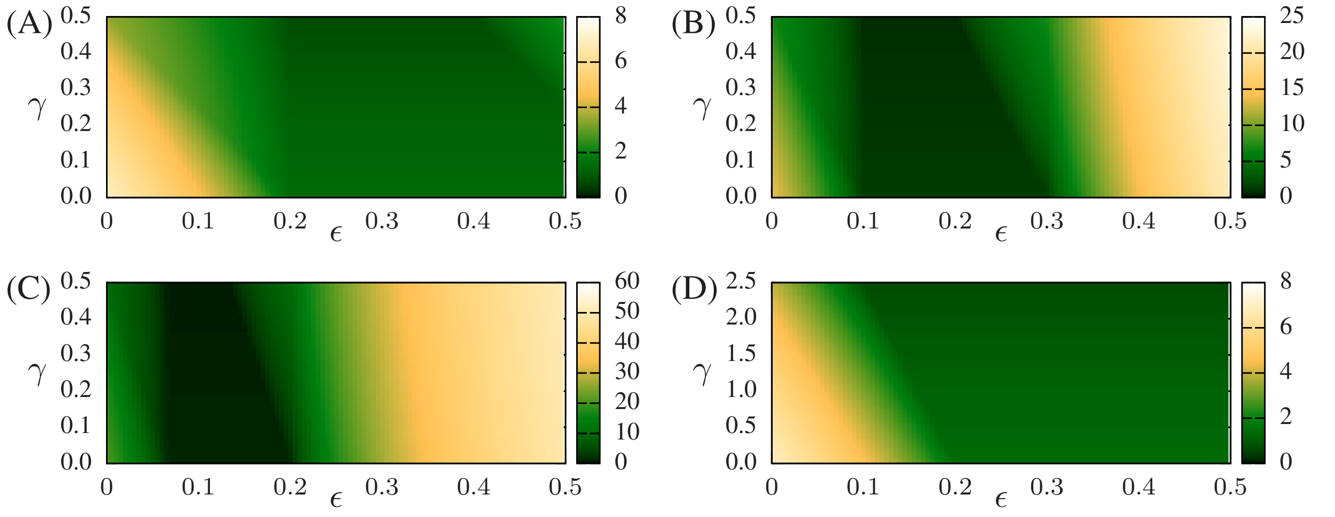

Figure 4. Sum of positive Lyapunov exponents for a network constructed by coupling two all-to-all subnetworks. N=10 and ℓ_{12}=5 in (A), N=20 and ℓ_{12}=10 in (B), N=30 and ℓ_{12}=15 in (C), and N=10 and ℓ_{12}=1 in (D).

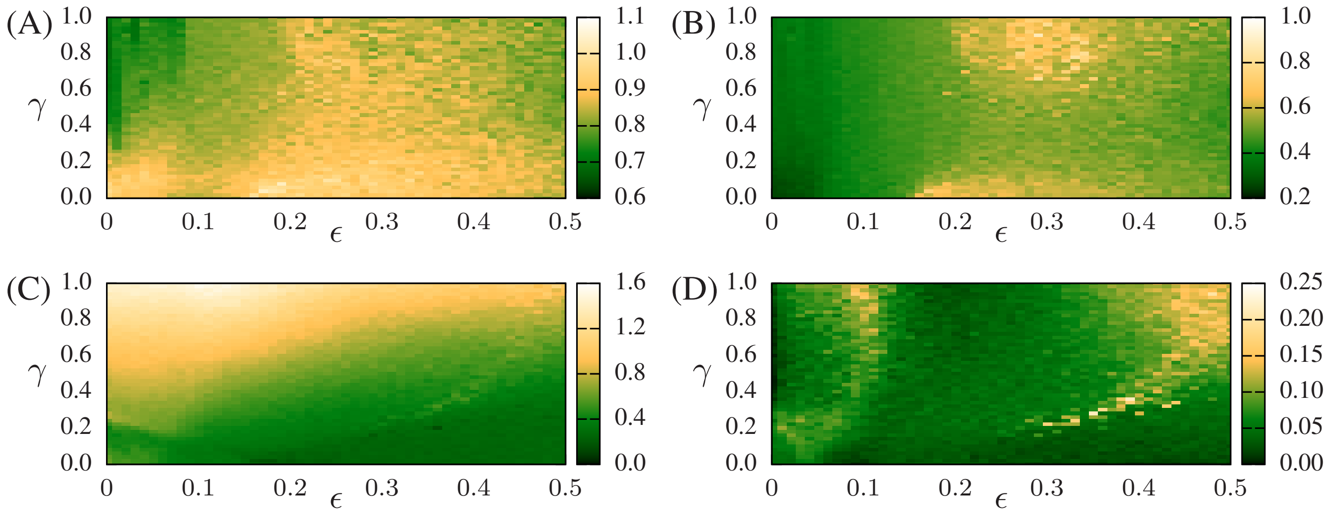

Figure 5. N=10 and L_{12}=5. Sum of positive Lyapunov exponents shown in (A) and (C), and I_C shown in (B) and (D), for two coupled stars of Hindmarsh-Rose neurons with nonlocal inter inhibitory coupling (A) and (C), and inter non-local excitatory coupling (B) and (D).

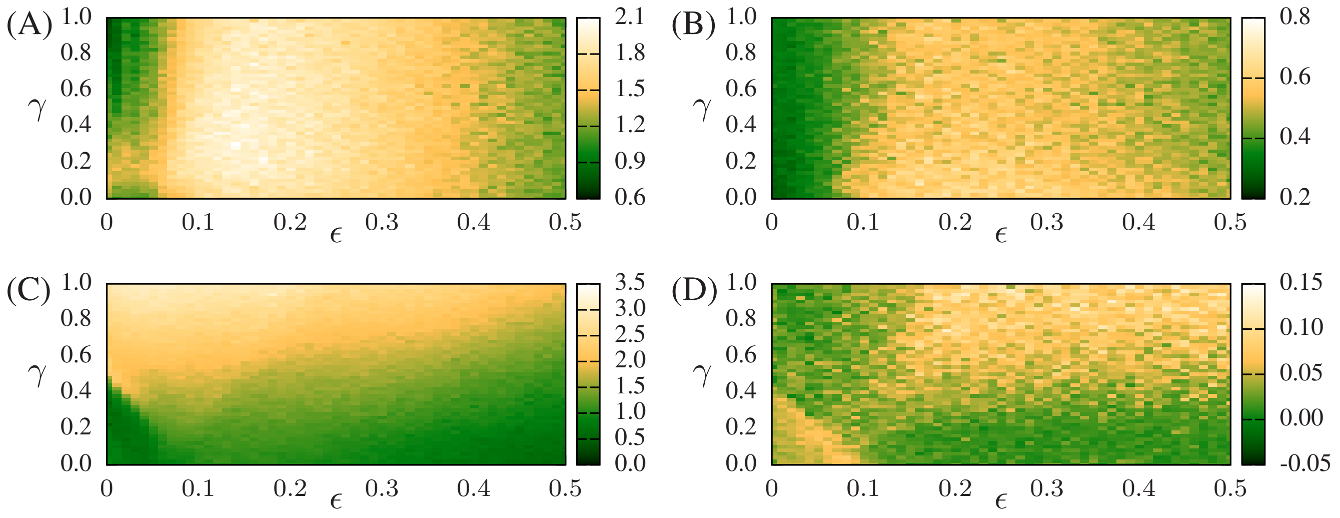

Figure 6. N=30 and ℓ_{12}=15. Sum of positive Lyapunov exponents shown in (A) and (C), and I_C shown in (B) and (D) for two coupled rings of Hindmarsh-Rose neurons with inter inhibitory coupling (A) and (B), and inter excitatory coupling (C) and (D).

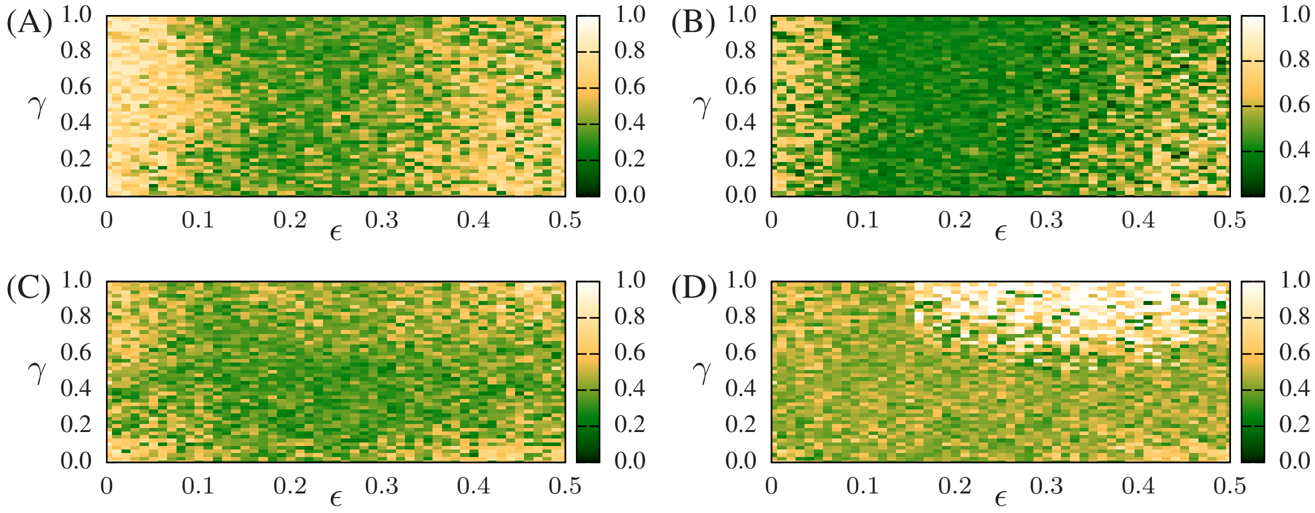

Figure 7. N=10 and ℓ_{12}=5. Order parameter r in (A) and (C), and δr shown in (B) and (D), for two coupled rings of Hindmarsh-Rose neurons with inter inhibitory coupling (A) and (B), and inter excitatory coupling (C) and (D).

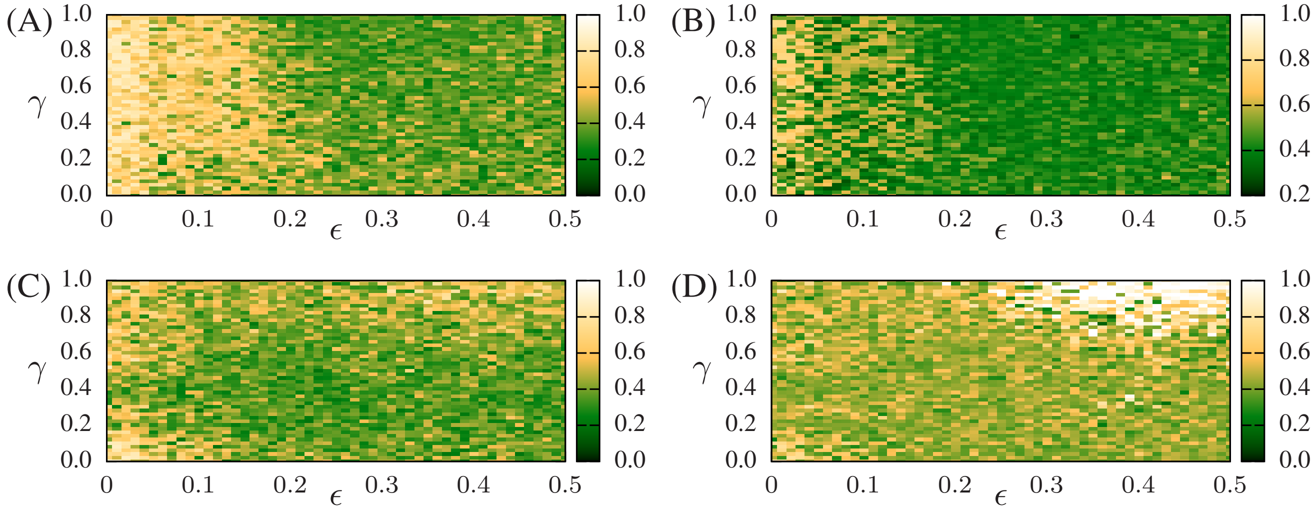

Figure 8. N=10 and ℓ_{12}=5. Order parameter r in (A) and (C), and δr shown in (B) and (D), for two coupled stars of Hindmarsh-Rose neurons with inter inhibitory coupling (A) and (B), and inter excitatory coupling (C) and (D).

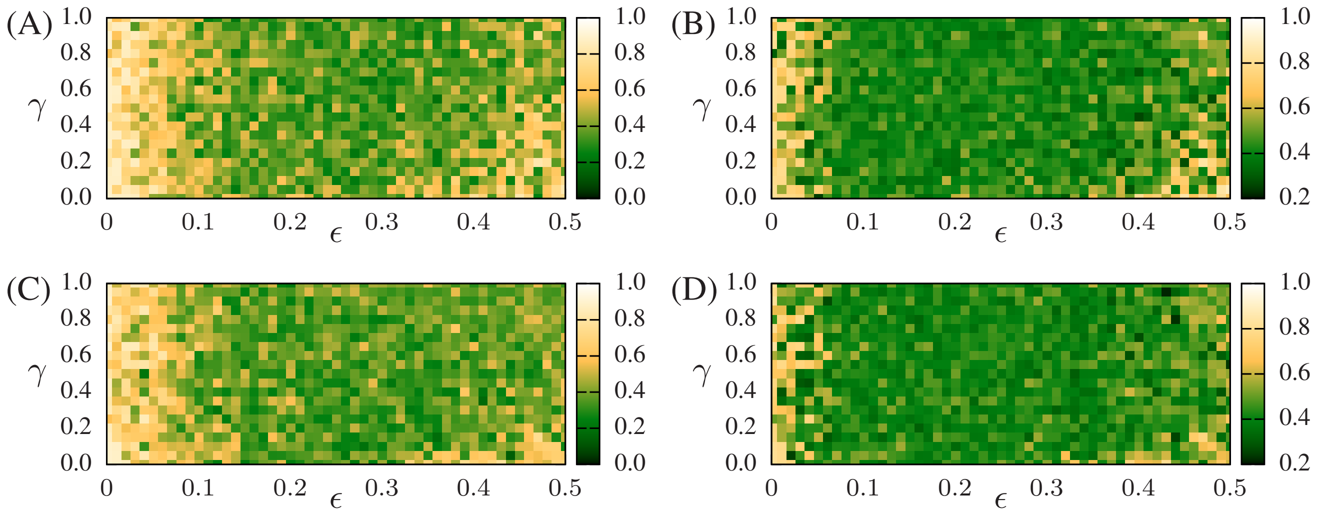

Figure 9. N=10 and ℓ_{12}=1. Order parameter r in (A) and (C), and δr shown in (B) and (D) for two coupled rings of Hindmarsh-Rose neurons with inter inhibitory coupling (A) and (B), and inter excitatory coupling (C) and (D).

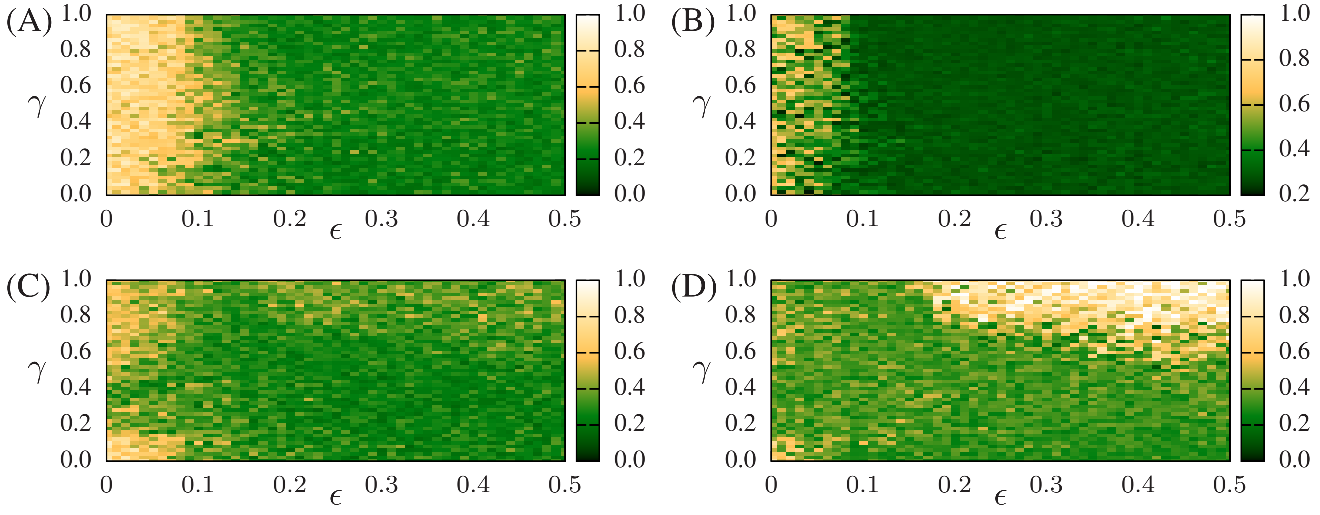

Figure 10. N=20 and ℓ_{12}=10. Order parameter r in (A) and (C), and δr shown in (B) and (D), for two coupled rings of Hindmarsh-Rose neurons with inter inhibitory coupling (A) and (B), and inter excitatory coupling (C) and (D).
